# High activity and specificity of bacteriophage cocktails against carbapenem-resistant *Klebsiella pneumoniae* belonging to the high-risk clones CG258 and ST307

**DOI:** 10.3389/fmicb.2024.1502593

**Published:** 2024-12-09

**Authors:** Sara Tellez-Carrasquilla, Lorena Salazar-Ospina, J. Natalia Jiménez

**Affiliations:** Grupo de Investigación en Microbiología Básica y Aplicada (MICROBA), Escuela de Microbiología, Universidad de Antioquia, Medellín, Colombia

**Keywords:** carbapenem-resistant, *Klebsiella pneumoniae*, bacteriophage, CG258, ST307, cocktails, high-risk clones

## Abstract

**Introduction:**

The widespread clinical and environmental dissemination of successful clones of carbapenem-resistant *Klebsiella pneumoniae* (CRKP) represents a serious global public health threat. In this context, lytic bacteriophages have emerged as a promising alternative for controlling these pathogens. This study describes the biological, structural, and genomic characteristics of lytic bacteriophages against the high-risk CRKP clones CG258 and ST307 and describes their performance in combination.

**Methods:**

An experimental study was carried out. Bacteriophages were isolated from hospital wastewater and from wastewater treatment plants (WWTP). Bacteriophages were isolated using the double layer agar technique and their characterization included host range (individual and cocktail), plating efficiency (EOP), infection or bacterial killing curve, one-step curve, bacteriophage stability at pH and temperature conditions, transmission electron microscopy (TEM) and whole genome sequencing.

**Results:**

After purification, five active bacteriophages against CRKP were obtained, three bacteriophages (FKP3, FKP4 and FKP14) had targeted activities against CG258 CRKP and two (FKP10 and FKP12) against ST307 isolates. Seven cocktails were prepared, of which Cocktail 2, made up of the bacteriophages FKP3, FKP10, and FKP14, showed the best activity against 85.7% (*n* = 36/42) of CRKP isolates belonging to both clones, CG258 (80.8%; *n* = 21/26) and ST307 (93.8%, *n* = 15/16). The efficiency of the plating (EOP), infection curve, and one-step growth curve showed that the cocktail phages efficiently infected other CRKP isolates (EOP ≥ 0.5), controlled bacterial growth up to 73.5%, and had short latency periods, respectively, (5–10 min). In addition, they were stable at temperatures between 4°C and 50°C and pH between 4 and 10. All bacteriophages belonged to the *Caudoviricetes* class, and no genes associated with virulence factors or antibiotic resistance were detected.

**Conclusion:**

These findings showed bacteriophages and phage cocktails with high specificity against CRKP belonging to the successful clones CG258 and ST307 with promising characteristics, making them an alternative for controlling these clones in different environmental or health settings, biocontrol agents, or disinfectants in industry and in the field of diagnosis.

## 1 Introduction

Carbapenem-resistant *Klebsiella pneumoniae* (CRKP) infections have a major clinical impact globally, given their high levels of multidrug resistance (MDR), increased mortality (33–50%), length of hospital stays, and high healthcare costs (Xu et al., 2017). Currently, this microorganism is considered an urgent priority by the Centers for Disease Control and Prevention (CDC) (2024) and the World Health Organization (2024).

Several CRKP clones have been described around the world, which differ according to geographical location. Among the most important clones, ST14, ST15, ST147, and ST101 have been reported with the highest frequency; however, the global spread of CRKP has been mainly linked to an expansion of successful clones belonging to CG258 (including ST258, ST512 and ST11) harboring principally KPC-type carbapenemase (*K. pneumoniae* carbapenemase) (Wyres et al., 2020; Lee et al., 2016). These clones account for 70–90% of all CRKP strains and are responsible for 68% of outbreaks in hospital settings. In addition, they have been reported to cause outbreaks in the United States, Canada, European, Asian, and Latin American countries (Wyres et al., 2020; Yang et al., 2021; Munoz-Price et al., 2013; Kitchel et al., 2009; Schwaber et al., 2011). In recent years, CRKP clones belonging to ST307 have successfully emerged in the United States, Italy, and Colombia owing to their virulence factors, which provide them with adaptive advantages in various scenarios (Villa et al., 2017; Bonnin et al., 2020; Peirano et al., 2020).

On the other hand, CRKP has spread worryingly to other settings, such as the community and the environment, because it harbors successful mobile genetic elements that confer resistance and easy dissemination and inappropriate use of antibiotics in diverse anthropogenic activities (Kahn, 2017). Several studies have described the presence of this microorganism in effluents from hospital wastewater and domestic wastewater treatment plants (WWTPs) (Surleac et al., 2020; Moges et al., 2014). In addition, these bacteria have been classified as emerging pollutants that persist in effluents because WWTPs are not designed to remove them, increasing the risk of dissemination and infection due to the use of these waters (Li et al., 2022).

Considering that therapeutic options are becoming scarce and that environmental biocontrol alternatives are necessary to contain this problem, strategies based on the use of lytic bacteriophages have been reconsidered in recent years (Hobson et al., 2022; Dancer, 2014). These viruses have a great potential to infect bacteria in a specific way; they do not affect other bacterial communities or eukaryotic cells, making them safe alternatives for humans and friendly to the environment (Sharma et al., 2017). Bacteriophages have a wide field of application, and there are currently a variety of studies evaluating their activity in different scenarios like compassionate therapy in humans, disinfectants in hospital settings, the removal of biofilms, and as an alternative biocontrol agent for the treatment of wastewater (McCallin and Zheng, 2019; Dedrick et al., 2023; Accolti et al., 2018; Ho et al., 2016; Łusiak-Szelachowska et al., 2020; Fu et al., 2010; Jassim et al., 2016). However, bacteriophage characterization processes based on biological, structural, and genomic analyses form the basis of application studies.

Numerous studies have been conducted to characterize bacteriophages that are active against antibiotic-resistant pathogens, specifically bacteria belonging to the ESKAPE group (*Enterococcus faecium*, *Staphylococcus aureus*, *K. pneumoniae*, *Acinetobacter baumannii*, *Pseudomonas aeruginosa* y *Enterobacter* spp.) (Yoon et al., 2013; Chen et al., 2021; Peng et al., 2014; Adnan et al., 2019; Zhao et al., 2019). However, studies of bacteriophages against specific *K. pneumoniae* clones have been limited and have focused on the isolation of active bacteriophages against other clones, such as ST11, ST16, and ST15 (Martins et al., 2022a; Horváth et al., 2023; Fang and Zong, 2022). Only a few of them are described in the characterization of bacteriophages active against ST258 (Tisalema-Guanopatín et al., 2023; Thiry et al., 2019; D’Andrea et al., 2017). In this context, this study describes the biological and structural characteristics of bacteriophages with high specificity against CRKP belonging to CG258 and ST307. In addition, their activity in combination was evaluated to obtain an effective alternative for controlling endemic clones disseminated worldwide.

## 2 Materials and methods

### 2.1 Specimen collection

Wastewater samples were collected from the effluents of a tertiary-care hospital (these effluents comprising wastewater from emergency rooms, hospitalization, intensive care unit, special care unit, internal medicine and food service) and from the affluents and effluents of a wastewater treatment plant (WWTP) in the city of Medellín (Colombia), between September 2021 and November 2022. Five hundred milliliters of each wastewater sample were collected and processed within the first 2–24 h to avoid alterations in the microbial communities (Van Charante et al., 2021).

### 2.2 Selection of host bacteria

Three carbapenem-resistant *K. pneumoniae* isolates harboring *bla-_KPC_* belonging to two successful clones were used as host bacteria for the search for bacteriophages. These included isolates from CG258 (ST512 *n* = 1 and ST258 *n* = 1) and ST307 (*n* = 1) (Supplementary Table S1). The bacterial isolates had clinical origins and were obtained from the Microbiological strain collection of the Grupo de Investigación en Microbiología Básica y Aplicada (MICROBA) (Ocampo et al., 2016; Cienfuegos-Gallet et al., 2017). The bacterial isolates were previously characterized, and identification and susceptibility tests were performed using a semi-automated method VITEK^®^ 2 Compact system (bioMérieux, Inc. Hazelwood, MO). In addition, genes encoding carbapenemases were identified by PCR and sequenced to determine gene-variants; further, molecular typing was performed using Multilocus sequence typing (MLST) (Ocampo et al., 2016; Poirel et al., 2011; Ellington et al., 2007; Diancourt et al., 2005).

### 2.3 Bacteriophages isolation

#### 2.3.1 Samples processing

Wastewater samples were processed with chloroform (Merck, KGaA, Darmstadt, Germany) to release the bacteriophages from organic matter (relation 1:10). Subsequently, the aqueous phase was recovered, centrifuged (4500 rpm for 10 min at 4°C), and filtered through 0.22 μm syringe filters (Minisart^®^ Sartorius AG, Germany) according to a modification to the protocol by Kropinski and Clokie (2009). To selectively increase the number of bacteriophages in the processed samples, enrichment was performed. The processed sample was placed in contact with each host bacterium in LB broth (Miller, Becton Dickinson Difco™) with CaCl_2_ (2 mM, Biobasic). Subsequently, samples were incubated at 35 ± 2°C for 24 h in a shaking humidified bath, centrifuged (4500 rpm for 10 min at 4°C), and filtered (0.22 μm syringe Filter, Minisart^®^ Sartorius AG, Germany) (Kropinski and Clokie, 2009).

#### 2.3.2 Bacteriophage detection and plaque purification

To detect bacteriophages in the enriched samples, the spot test method described by Clokie et al. was performed (Kropinski and Clokie, 2009). Subsequently, to obtain isolated plaques, positive samples were serially diluted (1:10) and seeded using the double-layer agar method (Adams, 1959). The plaques were selected based on size and translucency, and only one plaque was collected and deposited in Eppendorf tubes with 500 μL of SM buffer (100 mM, NaCl Merck Millipore; 50 mM Tris–HCl [pH 7.5]; 8 mM, MgSO_4_ Scharlau; 0.01% gelatin Oxoid). Subsequently, the plaques were mixed in SM buffer until homogeneous, and serial dilutions were made (1:10) and seeded using the double layer agar method (Adams, 1959). Each bacteriophage plaque was purified three times with repeated isolation to obtain a single bacteriophage.

#### 2.3.3 Concentration and quantification of bacteriophages

Bacteriophages were concentrated using the double-layer agar method (Swanstrom and Adams, 1951). Briefly, plaques in SM buffer obtained after purification were mixed and serially diluted (1:10). Subsequently, dilutions of higher phage concentrations (less diluted) were plated using the double-layer agar method. This procedure was repeated nine times until 10 replicates were completed. After incubation, the entire top-agar layer of the ten replicas was collected and added to 15 mL of SM buffer; it was then mixed, centrifuged (4500 rpm for 10 min at 4°C), and the supernatant was filtered (0.22 μm Minisart^®^ Syringe Filter) (Swanstrom and Adams, 1951). Finally, bacteriophage solutions were quantified twice on different days and each dilution was plated in duplicate using the double-layer agar method and stored in SM buffer at 4°C and at-80°C with 50% glycerol (Amresco, Inc., Solon Ohio, United States) (Kropinski and Clokie, 2009; Adams, 1959). The stability of the bacteriophages under these storage conditions is monitored over time.

### 2.4 Characterization of bacteriophages

#### 2.4.1 Host range evaluation

A host range was performed using 131 bacterial isolates to determine the specificity of bacteriophage infection. The evaluation was performed in triplicate using the spot test method, and plaque formation was verified by performing serial dilutions of the phages and plating in the quantitative spot test to confirm productive infection in the bacteriophage-susceptible isolates (Kropinski and Clokie, 2009). An inter-species and inter-genus assessment was performed on 31 isolates other than *K. pneumoniae*: *Klebsiella oxytoca* (*n* = 3)*, Citrobacter freundii* (*n* = 3)*, Enterobacter cloacae* (*n* = 3), *Escherichia coli* (*n* = 5), *Serratia* spp. (*n* = 4), *Pseudomonas aeruginosa* (*n* = 3)*, Acinetobacter baumannii* (*n* = 3)*, Aeromonas* SPP (*n* = 2)*, Ralstonia paucula* (*n* = 2), and *Staphylococcus aureus* (*n* = 3). Additionally, the intra-species host range was determined in 100 isolates of *K. pneumoniae*: 25 carbapenem-susceptible and 75 carbapenem-resistant KPC (*bla*_-KPC-2_ *n* = 45 and *bla*_-KPC-3_ *n* = 30), belonging to CG258 (*n* = 25), ST307 (*n* = 15), ST14 (*n* = 10), and other STs (*n* = 25) (Supplementary Table S2). The CRKP isolates were also resistant to other antibiotic families (Supplementary Table S3), such as aminoglycosides (57.9%, *n* = 44/76), quinolones (82.14%, *n* = 23/28), fluoroquinolones (72.7%, *n* = 56/77), glycylcycline (67.2%, *n* = 39/58), and nitrofurans (100%, *n* = 10/10). The bacterial isolates were obtained from the strain collection of the Grupo de Investigación en Microbiología Básica y Aplicada (MICROBA).

#### 2.4.2 Efficiency of plating

Efficiency of plating was assessed for bacteriophage-susceptible *K. pneumoniae* isolates identified in the host range. This assessment was conducted using the quantitative spot test to determine the efficiency of phage infection against different *K. pneumoniae* isolates by comparing plaque production (Kropinski and Clokie, 2009). The EOP was calculated by dividing the number of plaques produced by the evaluated bacteria by the number of plaques produced by the host bacterium. Efficiency of plating values ≥0.5 were considered an efficient infection, values between 0.1 and less than 0.5 were considered a moderately efficient infection, values between 0.001 and less than 0.1 an infection with low efficiency, and values <0.001 were an inefficient infection (Khan Mirzaei and Nilsson, 2015). Each experiment was conducted in triplicate.

#### 2.4.3 Preparation and evaluation of bacteriophage cocktails

Different bacteriophage cocktails were prepared using combinations of 2 until 4 phages, each at a final concentration of 3×10^7^ UFP/ml, according to previous studies to prevent aggregation among bacteriophages (Zurabov et al., 2023; Asghar et al., 2022). The selection of the combination of bacteriophages for each cocktail was based on host range and EOP results. Cocktail assessments were performed using the same strains used in the intraspecies host range. First, the activity of the cocktails was determined using a spot-test. Then, serial dilutions of the cocktails were performed and seeded using the quantitative drop test to confirm productive infection in cocktail-susceptible isolates (Kropinski and Clokie, 2009). Finally, the best performing cocktail was selected, and individual characterization (biological, structural, and genomic) of the cocktail phages was performed. All experiments were performed in triplicate.

#### 2.4.4 Infection or killing curve

To evaluate the control of bacteriophage on bacterial growth, the host bacteria in exponential phase (approximate concentration of 1.5–5×10^8^ CFU/ml) were placed in contact with different concentrations of the phage (MOI 1, 0.1, and 0.01). The experiment was conducted on 96-well plates, and absorbance readings were taken every hour for 23 h at 600 nm using a Multiskan GO Microplate Spectrophotometer, using SkanIt™ Software (Thermo Scientific, v6.1.1) (Martins et al., 2022b). Additionally, the effects of the cocktail at different concentrations (MOI 1, 0.1, 0.01) on the inhibition of bacterial growth were evaluated. Each experiment was conducted in triplicate.

#### 2.4.5 Adsorption time and one-step growth curve

To define the adsorption time, the host bacterium in the exponential phase (1×10^8^ CFU/ml) was placed in contact with the bacteriophage at an MOI of 0.01 (1×10^6^ PFU/ml), followed by incubation at 37°C in a shaking humidified bath. Subsequently, samples were collected every 5 min for 20 min. Each sample was filtered, and serial dilutions (1:10) were performed to quantify the number of phages each time using the double-layer agar technique (Adams, 1959). To obtain the one-step curve, adsorption was first performed. The phages (1×10^6^ UFP/ml) were placed in contact with host bacteria in the exponential phase (1×10^8^ CFU/ml) at an MOI of 0.01 and incubated for 10 min at 37°C. After this time, it was centrifuged (4500 rpm for 10 min at 4°C), and the supernatant was discarded; the pellet was re-suspended in the same volume of broth and incubated at 37°C. Two samples were taken every 5 min for 45 min; one of them was treated with chloroform (1:10 ratio) to determine the eclipse time, and the other sample was used to determine the latency time (Zhao et al., 2019). Both samples were filtered and serially diluted to quantify the number of bacteriophages each time using the double-layer agar method (Adams, 1959). The burst size was determined by dividing the average number of viral particles produced by the number of infected cells (D’Andrea et al., 2017). The experiments were conducted in triplicate.

#### 2.4.6 Susceptibility to different pH and temperature conditions

Each bacteriophage at a concentration of 1×10^6^ PFU/ml was exposed to temperatures of 4°C, 25°C, 37°C, 50°C, 60°C, and 70°C to determine temperature susceptibility. After 1 h of exposure, serial dilutions (1:10) were prepared and plated using the quantitative spot test (Kropinski and Clokie, 2009). To assess pH sensitivity, the bacteriophages were diluted in SM buffer adjusted to different pH (CDC, 2024; Wyres et al., 2020; Yang et al., 2021; Munoz-Price et al., 2013; Kitchel et al., 2009; Villa et al., 2017; Peirano et al., 2020) to reach a final concentration of 1×10^6^ PFU/ml. After 1 h of exposure, serial dilutions (1:10) were prepared and plated using the quantitative spot test (Kropinski and Clokie, 2009). The experiments were conducted in triplicate.

#### 2.4.7 Transmission electron microscopy

The bacteriophages (1 × 10^10^ UFP/ml) were purified by ultracentrifugation and washed with ammonium acetate (Merck KGaA, Darmstadt, Garmany) according to Kropinski and Clokie (2009). To obtain transmission electron micrographs of the phages, a drop of each high-titer phage was deposited on a carbon-coated Formvar layer held by a copper grid. The samples were allowed to dry for 30 min, and excess liquid was removed. Finally, the phages were negatively stained with 2% phosphotungstic acid and examined under a transmission electron microscope (Domingo-Calap et al., 2020). All measurements were performed using Image J program v1.51 2018. Three different particles of which phage were measured.

#### 2.4.8 Genetic material extraction and sequencing

A solution of bacteriophages at a concentration of 10^10^ PFU/ml was used, which was enzymatically digested with RNAse (Sigma-Aldrich, St. Louis, United States) and DNAse I (Thermo Scientific, Massachusetts, United States) overnight at 37°C. After incubation, the enzymes were inactivated at 80°C for 15 min. Subsequently, a proteolysis buffer was added [final concentration: Proteinase K (50 μg/mL) (Thermo Scientific, Vilnius, Lithuania), EDTA pH8 (20 nM) (Honeywell, Wunstorfer, Germany), and SDS (0.5%) (Merck KGaA, Darmstadt, Garmany)], followed by incubation at 56°C for 1 h, and extraction was continued using the phenol-chloroform protocol with some modifications (Payaslian et al., 2021). The genetic material was quantified by fluorometry using Qubit (Life Technologies, Singapore) and Picogreen (Quant-iT™ PicoGreen™ Life Technologies, Oregon, United States). Genome quality and integrity were assessed using NanoDrop (Thermo Scientific, United States) and Agilent genomic DNA screen tape (DIN, DNA integrity number). Phage genomic DNA libraries and sequencing were performed by Psomagen Inc. (Rockville, Maryland, United States). Briefly, DNA libraries were prepared using an Illumina Truseq DNA PCR-free (350 bp insert) Library Prep Kit (Illumina, California, United States). The libraries were sequenced on the Illumina NovaSeq 6000 S4 platform with 151 bp paired end reads (2×151), with an approximate yield of 24 million reads per sample.

#### 2.4.9 Genome assembly and annotation

Quality control of the raw sequences was performed using FastQC v0.12.1. Low-quality bases (Phred <30), adapters, and duplicate sequences were removed using FastP v0.23.4, and Trimmomatic v0.39 (Chen et al., 2018; Bolger et al., 2014). Subsequently, contaminated sequences were removed from the Illumina sequencing vector phage PhiX174 (NC_001422.1), and from the phage host *K. pneumoniae* F17KP0040 (GCA_012971225.1) using BBDuk v39.01 from the BBMap suite tools^1^ (Mukherjee et al., 2015). The filtered readings were assembled using SPAdes v3.15.5 (Prjibelski et al., 2020). The resulting contigs were lined with *K. pneumoniae* phages previously reported using BLASTn,^2^ and the readings that were mapped to these counts using BBDuk were retrieved and assembled *de novo* using SPAdes. Phageterm v3.0.1 was used for the prediction of physical endings and the rearrangement of phage genomes (Garneau et al., 2017). Complete, high-quality genomes were obtained using Pilon v1.24, and genome quality and integrity were assessed using QUAST v5.2.0 and CheckV v.1.0.1 (Walker et al., 2014; Gurevich et al., 2013; Nayfach et al., 2021). Genome annotation was performed using Pharokka v1.7.1 (Bouras et al., 2023). Briefly, coding sequences (CDS) were predicted using PHANOTATE v1.5.1, tRNAs were predicted using tRNAscan-SE v2.0.12, mRNAs were predicted using Aragorn, and CRISPR sequences were predicted using CRT. Functional annotation was performed by searching the PHROGS database for CDS using MMseqs2 and PyHMMER (Terzian et al., 2021; Steinegger and Söding, 2017; Larralde and Zeller, 2023). Virulence factors and resistance genes were predicted using the VFDB databases and CARD (Chen et al., 2005; Alcock et al., 2020). Contigs were matched to their closest hit in the INPHARED database using mash (Cook et al., 2021; Ondov et al., 2016). The annotation was improved using Phold v0.1.3,^3^ a bacteriophage genome annotation tool based on protein structural homology. Finally, the annotation of the phage replicative cycle was predicted *in silico* using BACPHLIP, PhageAI v.1.0.0, and phaTYP (Hockenberry, 2021; Tynecki et al., 2020; Shang et al., 2023). The bacteriophage genomes were submitted to the National Center for Biotechnology Information (NCBI) database. The genome figures were created in PATRIC (Davis et al., 2019).

#### 2.4.10 Comparative genomics and phylogenetic analysis

First, the genomic similarity of the phages was compared using ProgressiveMauve (Darling et al., 2010). Closely related phages were identified using BLASTn on the GenBank NCBI virus database. Then, the average nucleotide identity (ANI) was calculated based on BLAST + (ANIb) by comparing the three phage genomes to those with highest score and identify (> = 90%) using JSpeciesWS (Richter et al., 2016). Based on this comparison, closely related phages with the highest ANIb values and other dsDNA phages were used to classify the phages to family levels using a proteome-based clustering strategy on the ViPTree server (Nishimura et al., 2017). Phages with the highest VipTree tBLASTx scores (S_G_) and outgroups from the *Autographiviridae* and *Drexleviridae* families were selected to perform a genome-genome distance phylogenetic analysis of phages using the Virus Classification and Tree Building Online Resource (VICTOR) (Meier-Kolthoff and Göker, 2017).

#### 2.4.11 Statistical analysis

The host range and cocktail evaluation results were described using absolute and relative frequencies. Quantitative variables were described using mean and standard deviation, and the assumption of normality was evaluated using Shapiro–Wilk. The analysis of quantitative variables was performed according to the assumption of normality; parametric tests included analysis of variance (ANOVA) or Student’s *t*-test, and nonparametric tests included the Kruskal–Wallis test or Wilcoxon test. The efficiency of plating results were classified according to the M. Khan Mirzaei et al. criteria (Khan Mirzaei and Nilsson, 2015). Values of *p* < 0.05 were considered statistically significant. The obtained information was analyzed using R studio v 2023.09.1 + 494.

## 3 Results

### 3.1 Bacteriophage isolation

**Figure 1:**
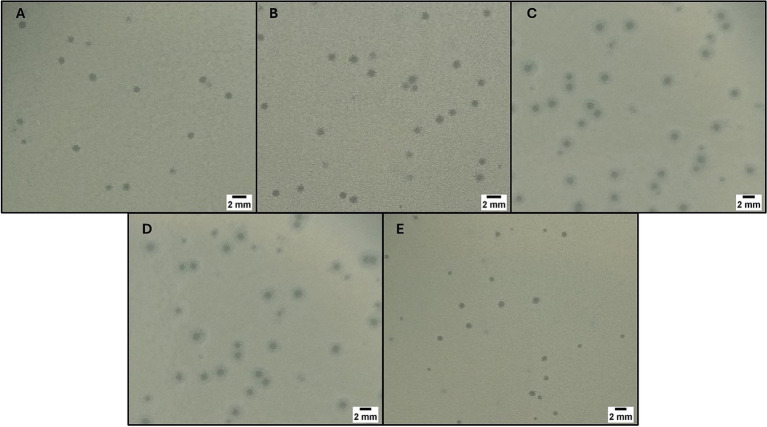
Bacteriophage plaque morphology. The figure shows the morphology of the plaques obtained using double-layer agar for the phages FKP3 **(A)**, FKP4 **(B)**, FKP10 **(C)**, FKP12 **(D)** and FKP14 **(E)**. The size of the plaques was between 1 and 2 mm, and FKP10 and FKP12 phages had a double halo.

Fourteen wastewater samples were collected, including 10 from hospital effluents and 4 from WWTPs (affluent *n* = 1, effluent *n* = 3). In total, 22 plaques of different morphologies were collected using the double-layer agar technique. After plaque purification process, 5 bacteriophages with large, sharp-edged, and translucent plaques were selected. Of the five isolated bacteriophages, three had the host bacterium CRKP belonging to CG258 (ST512, *n* = 2 and ST258, *n* = 1) and were named FKP3, FKP4, and FKP14. On the other hand, the remaining two bacteriophages had CRKP of ST307 as host bacteria and were designated FKP10 and FKP12. Bacteriophages produced plaques between 1 and 2 mm in diameter; in addition, the plaques produced by the bacteriophages FKP10 and FKP12 formed a double halo of inhibition (Figure 1).

### 3.2 Characterization of bacteriophages

#### 3.2.1 Host range evaluation

**Figure 2:**
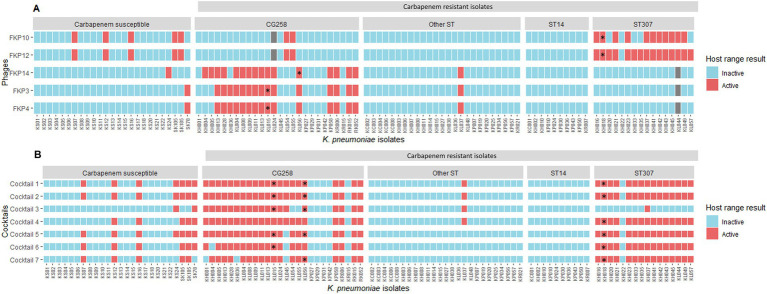
Intraspecies host range using individual bacteriophages and cocktails. **(A)** Shows the intraspecies host range of individual bacteriophages. * Host bacteria from which the bacteriophages were isolated: FKP3, FKP4, and FKP14 were obtained from CG258 isolates, whereas FKP10 and FKP12 were obtained from ST307 isolates. Gray squares correspond to strains against which the bacteriophages were not evaluated. **(B)** Presents the results of the intra-species host range of the evaluated cocktails. Considering that the individual host range showed high specificity for bacteriophages against some clones, bacteriophage combinations were performed to evaluate their performance in combination. Because the bacteriophages FKP3 and FKP4 had the same host range, one of them was selected to perform the combinations, except for Cocktail 3.

The evaluation of the inter-species and inter-genus host range showed that the isolated bacteriophages were not active against any of the bacterial strains of other genus and species evaluated (*n* = 0/31). Regarding the evaluation of intra-species strains (*K. pneumoniae*), bacteriophages showed lytic activity between 16 and 21%; the bacteriophage FKP12 had the highest activity by lysing 21 of the 100 *K. pneumoniae* isolates evaluated (Figure 2A). Bacteriophages were mainly active against *K. pneumoniae* isolates belonging to the same clonal group as their host bacterium. Bacteriophages FKP3, FKP4, and FKP14 obtained from CG258 host bacteria were active against 56% (14/25) and 60% (15/25) of CRKP of the same clonal group (CG258). Likewise, the FKP10 and FKP12 bacteriophages isolated from *K. pneumoniae* from ST307 were active against 66.7% (10/15) and 93.3% (14/15) of isolates belonging to the same ST of the host bacteria, respectively. Regarding the activity of phages in carbapenem-susceptible strains, FKP10 and FKP12 exhibited activity against 20% (*n* = 5/25) of the strains compared with FKP3, FKP4, and FKP14 phages, which exhibited activity against 4% (*n* = 1/25). In general, bacteriophages showed no activity against the CRKP strains of other STs, except for FKP10 and FKP12, which exhibited activity against ST231.

#### 3.2.2 Efficiency of plating

**Figure 3:**
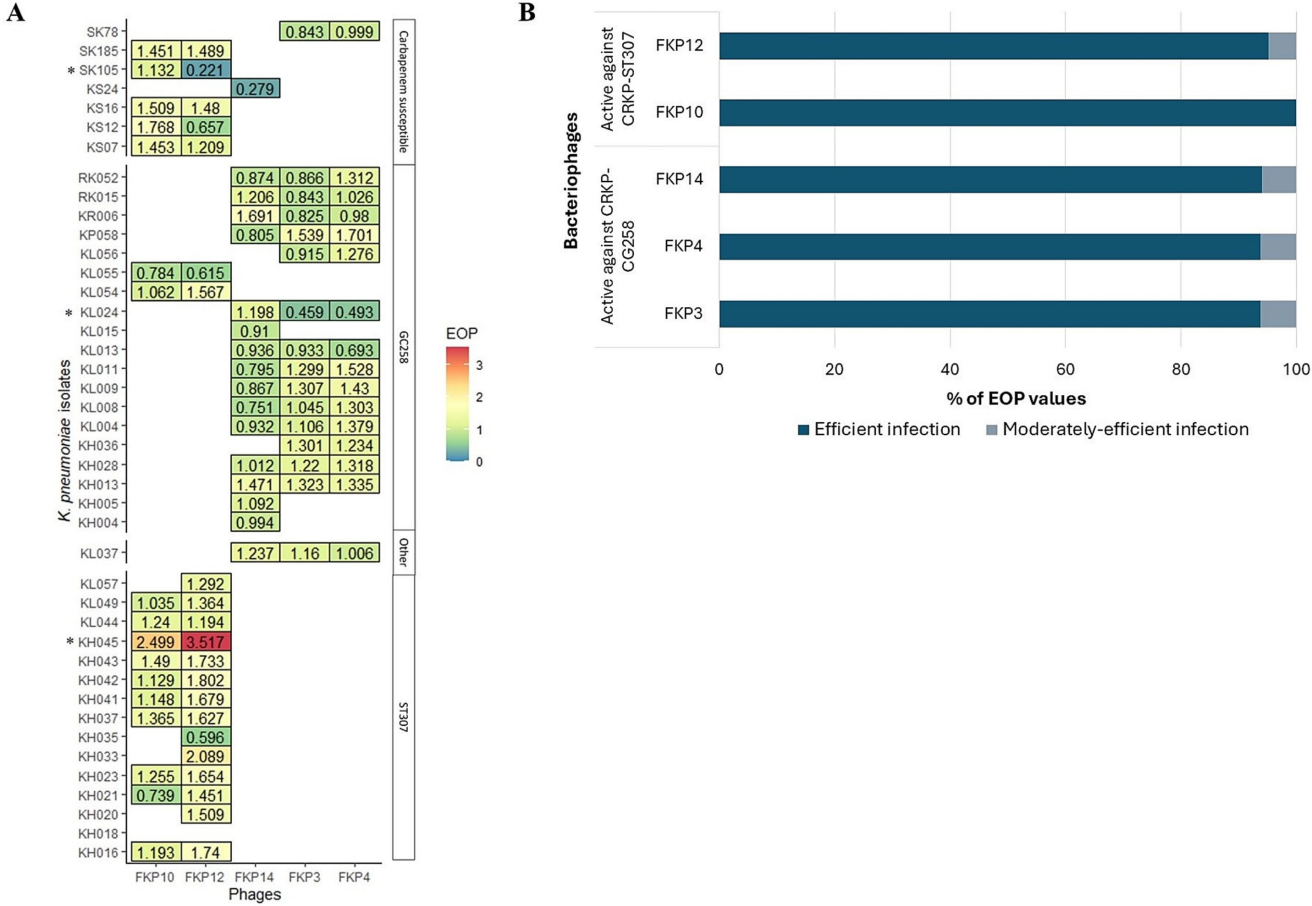
Efficiency of plating (EOP) of CRKP bacteriophages. **(A)** Shows the efficiency of plating evaluated in susceptible isolates in the host range. EOP values ≥ 0.5 indicate efficient infection; 0.1 ≤ EOP < 0.5, moderately efficient infection; and 0.001 ≤ EOP < 0.1, infection with low efficiency. * Bacteriophage FKP3 exhibited reduced infection efficiency with the KL024 isolate (EOP = 0.459; *p* < 0.05). The same result was observed for the FKP12 bacteriophage with isolate SK105 (EOP = 0.221; *p* < 0.05). On the other hand, the bacteriophages FKP10 (EOP = 2.499; *p* < 0.001) and FKP12 (EOP = 3.517; *p* < 0.001) exhibited statistically significant high infection efficiency with the KH045 isolate. **(B)** Presents the percentage of efficient and moderate infection values observed in bacteriophages.

The efficiency of the plating results is shown in Figure 3. In general, bacteriophages efficiently infected other *K. pneumoniae* isolates, when compared to infection against their host bacteria (EOP ≥ 0.5) (Figure 3A). FKP3, FKP4, and FKP14 phages efficiently infected 93.75% (*n* = 15), 93.75% (*n* = 15), and 94.1% (*n* = 16) of the isolates evaluated (Figure 3B), with EOP averages of 1.06 ± 0.27, 1.19 ± 0.31, and 1.00 ± 0.31, respectively. These three bacteriophages did not differ in the production of plaques (*p* = 0.204) (Figure 3A). On the other hand, the FKP10 and FKP12 bacteriophages efficiently infected 100% (*n* = 17) and 95.2% (*n* = 20) of the evaluated strains, respectively (EOP ≥ 0.5) (Figure 3B). In addition, they had EOP averages of 1.31 ± 0.40 and 1.45 ± 0.66, which did not differ significantly (*p* = 0.15) (Figure 3A). Finally, moderately efficient infection (0.1 ≤ EOP < 0.5) was observed in 6.25% (*n* = 1), 6.25% (*n* = 1), 5.9% (*n* = 1), and 4.8% (*n* = 1) of the isolates with the bacteriophages FKP3, FKP4, FKP14, and FKP12, respectively; further, low infection efficiency (0.001 ≤ EOP < 0.1) and inefficient infection (< 0.001) were not observed (Figure 3B).

#### 3.2.3 Preparation and evaluation of bacteriophage cocktails

In total, seven cocktails were prepared, five aimed at increasing the host range against clones belonging to CG258 and ST307 (Cocktails 1, 2, 5, 6, and 7), one aimed at isolates of ST307 (Cocktail 4), and another aimed at isolates of CG258 (Cocktail 3). The conformation of the cocktails is described in Table 1.

**Table 1:** Conformation of bacteriophages cocktails against *K. pneumoniae*.

	Bacteriophages				
Cocktails	Against CRKP of CG258			Against CRKP of ST307	
	FKP3	FKP4	FKP14	FKP10	FKP12
Cocktail 1	X		X		X
Cocktail 2	X		X	X	
Cocktail 3	X	X	X		
Cocktail 4				X	X
Cocktail 5	X		X	X	X
Cocktail 6	X			X	X
Cocktail 7			X	X	X

CRKP, carbapenem resistant *Klebsiella pneumoniae*.

The evaluation of the intra-species host range of the cocktails showed a broadening of the host range up to 43.1% (*n* = 44/102) compared with the values of the individual host range, which reached 21%. Furthermore, all seven cocktails showed an increase in activity for both CRKP isolates belonging to CG258 and ST307 (Figure 2B). Cocktails 1, 2, and 4 were active against 85.7% (*n* = 36/42; CG258 80.7%, *n* = 21/26; ST307, 93.75%, *n* = 15/16) of the isolates belonging to both clones. Cocktail 4, prepared to increase activity only against ST307 isolates, expanded the host range against CG258 isolates (85.7%; *n* = 36/42; CG258 *n* = 21; ST307 *n* = 15); while Cocktail 3, targeting CG258 strains, maintained its specificity against this same clonal group (47.6%; *n* = 20/42; CG258 *n* = 19; ST307 *n* = 1). Finally, Cocktails 1, 2, and 5 were active against 28% (*n* = 7) carbapenem-susceptible isolates, followed by cocktails 6 and 7 with 24% (*n* = 6) and cocktails 3 and 4 active against 8% (*n* = 2) and 20% (*n* = 5), respectively.

The results of the intra-species host range using cocktails (using the spot test) were confirmed using the plaque count to identify productive infection. These results allowed us to determine low plaque production of Cocktail 4, compared to Cocktails 1 and 2. Finally, of the seven cocktails prepared, cocktail 2, composed of the bacteriophages FKP3, FKP10, and FKP14, showed the best performance considering its high specificity for both CG258 and ST307 isolates (80.8 and 93.75%, respectively). Furthermore, the bacteriophages in this cocktail had higher EOP results. Phages from Cocktail 2 were then selected for individual characterization (biological, structural, and genomic).

#### 3.2.4 Infection or killing curves of individual phages and cocktail

**Figure 4:**
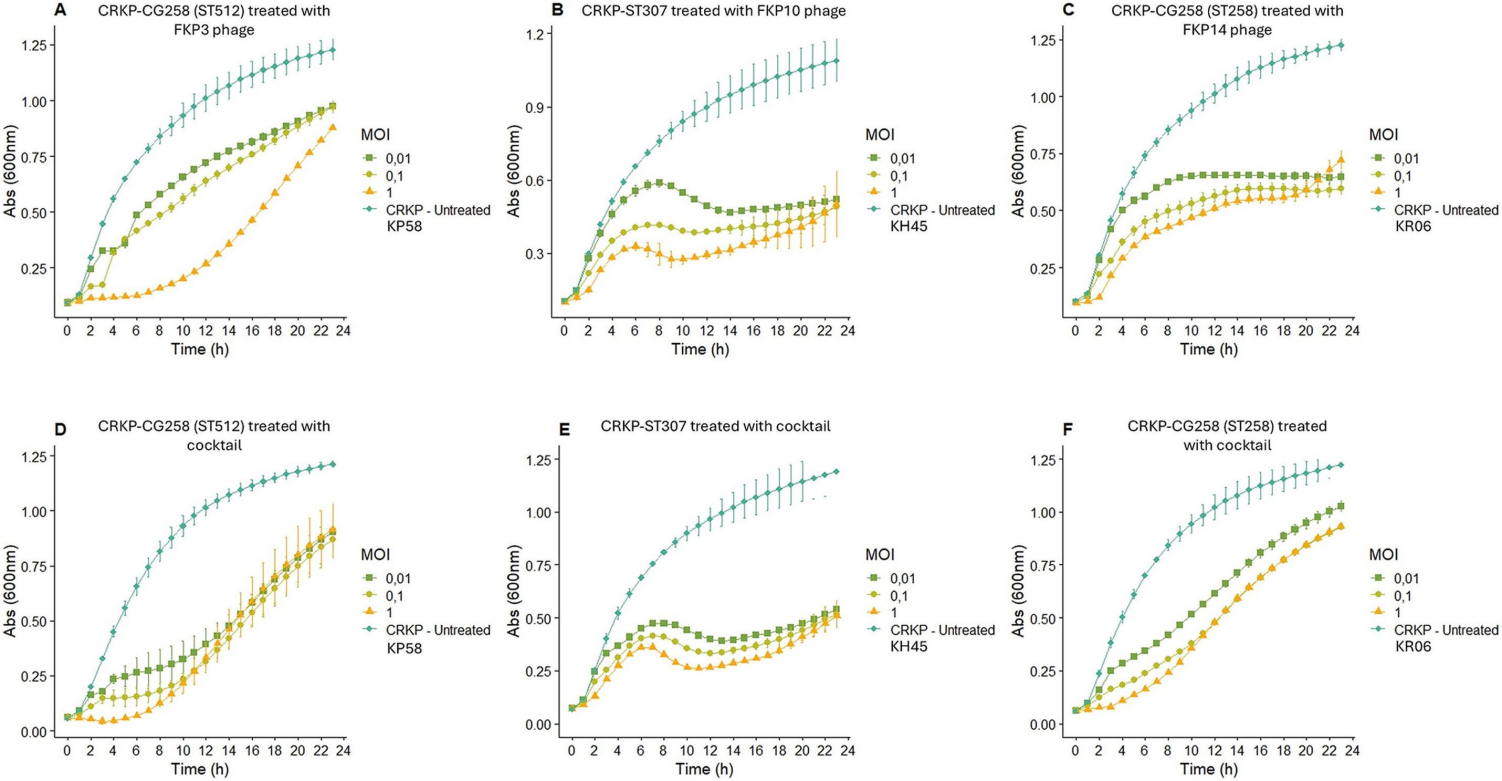
Bacterial infection or elimination curve. **(A–C)** Show the elimination curve of bacteriophage FKP3, FKP10 and FKP14, respectively. **(D–F)** Show the elimination curve of cocktail 2 (conformed for the bacteriophages FKP3, FKP10 and FKP14) with the host bacteria of each bacteriophage that composed that cocktail. MOI: multiplicity of infection.

Bacteriophage FKP3 showed a greater reduction in bacterial growth (82.70%) after 6 h of treatment at an MOI of 1, whereas lower MOIs (0.1 and 0.01) showed reduced effectiveness (Figure 4A). Overall, significant control of bacterial growth of the FKP3 phage was observed at 6, 12, and 23 h; however, the MOI of 1 remained superior throughout treatment (Figure 4A). On the other hand, when the selected cocktail 2 was used in the host strain of the FKP3 phage [CRKP-ST 512 isolate (KP58)], the MOI of 1 showed a similar behavior to that observed with the individual phage (MOI 1), but the cocktail 2 at lower MOIs performed better between hours 6 and 12 (MOI 0.1 69.02 to 77.63%; MOI 0.01 59.91 to 65.25%), showing a greater reduction than the individual phage (MOI 0.1 36.98 and 42.88%; MOI 0.0128.6 to 32.69%) (Figures 4A,D).

When the performance of phage FKP10 was evaluated against the CRKP-ST307 isolate (KH45), it was observed that the phage exerted maximum control of bacterial growth at 12 (67.55%), 18 (58, 65%), and 20 (52.73%) hours at MOIs of 1, 0, 1, and 0.01, respectively (Figure 4B). No differences were observed in the control of bacterial growth at the different MOIs, and a constant growth control was observed with the MOI of 1 (between 8 to 20 h; 61.3 to 67.5%), 0.1 (between 10 to 23 h, 53.32 to 58.65%), and 0.01 (between 14 to 23 h, 50.84 to 52.73%) (Figure 4B). In general, the use of the cocktail showed no difference in the control of bacterial growth at MOI of 1 and 0.1 compared with that observed for the individual phage; however, the cocktail at an MOI of 0.01 improved performance between hours 4 and 11 (Figure 4E).

Finally, the bacteriophage FKP14 evaluated against the CG258 isolate (KR06), maintained its highest peak activity at 2 (61.43%), 22 (51.6%) and 23 (47.28%) hours after treatment, for MOI of 1, 0.1, and 0.01, respectively, (Figure 4C). Likewise, a significant growth control was observed with the 3 MOI at 6, 12, and 23 h, and no differences were observed between phage activity at each MOI. On the other hand, the activity of the phage at different MOIs was constant over time (MOI 1; reduction from 47.46 to 53.14%; MOI 0.1 reduction from 40.40 to 51.6%; MOI 0.01, reduction from 40.87 and 47.28%) (Figure 4C). In contrast, the cocktail did not show a better performance than that observed with the individual bacteriophage; however, the cocktail exhibited a significant reduction in bacterial growth at 6, 12, and 23 h (Figure 4F).

#### 3.2.5 Adsorption time and one-step growth curve

**Figure 5:**
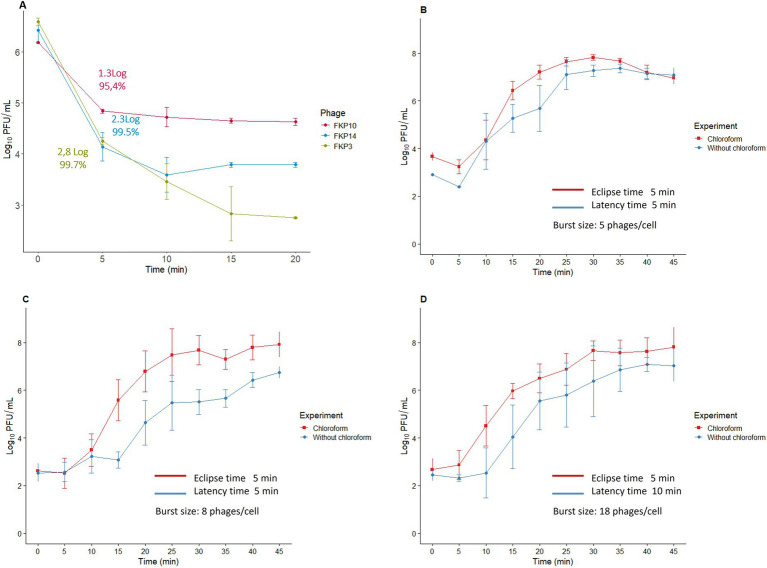
Characterization of bacteriophage replication cycle. This figure shows the results of the adsorption time **(A)**, and the one-step curve for the bacteriophages FKP3 **(B)**, FKP10 **(C)**, and FKP14 **(D)**.

The adsorption time of the three bacteriophages was 5 min, during which 95–99% of the viral particles were adsorbed (Figure 5A). The one-step curve showed that the bacteriophage replication times were between 30 and 45 min (Figures 5B–D); in addition, we found eclipse times of 5 min for the three bacteriophages and latency periods of 5, 5, and 10 min for FKP3, FKP10, and FKP14, respectively. Finally, a burst size of 5 PFU/cell was observed for phage FKP3, 8 PFU/cell for phage FKP10, and 18 PFU/cell for phage FKP14 (Figures 5B–D).

#### 3.2.6 Susceptibility to different pH and temperature conditions

**Figure 6:**
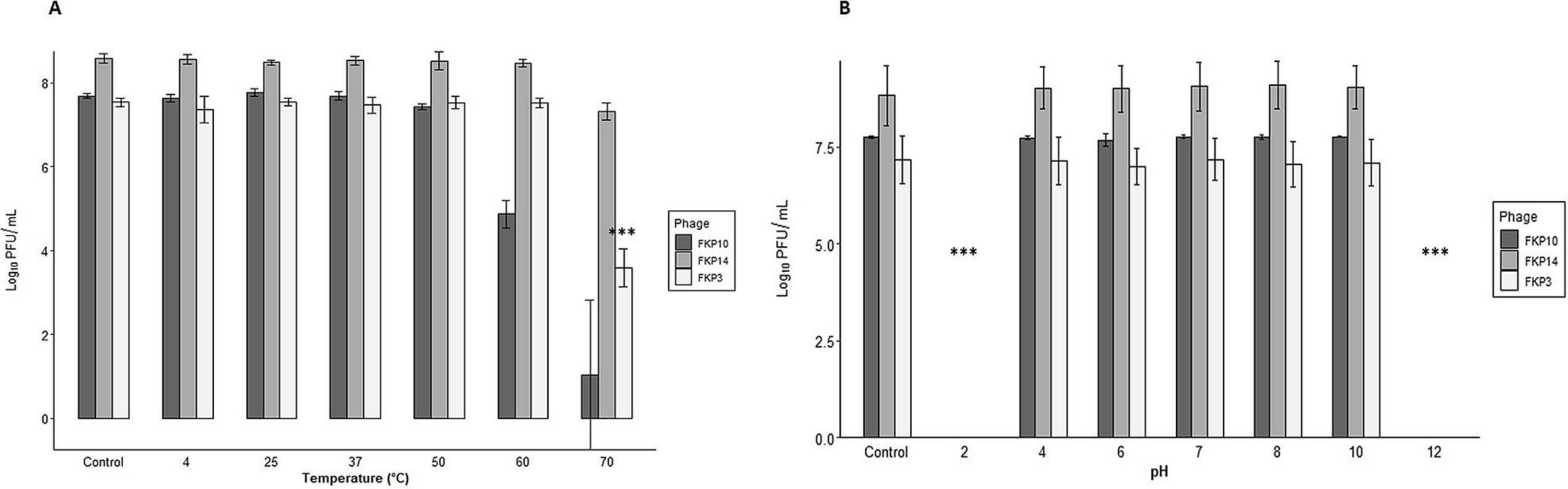
Characterization of bacteriophage susceptibility to pH and temperature conditions. **(A,B)** Show the results of phage stability at different temperatures and pH conditions, respectively. In general, all three bacteriophages were stable at temperatures between 4°C and 50°C. A decrease of 3.9 Log was observed in the FKP3 bacteriophage at a temperature of 70°C (ANOVA, *p* = 4.22×10^−11^ ***). A decrease of 2.8 Log at 60°C was observed in phage FKP10 (Kruskal-Wallis, Dunnet test adjusted with the Bonferroni method; *p* = 1.0) and 6.6 Log at 70°C (Kruskal-Wallis, Dunnet test adjusted with Bonferroni method; *p* = 0.258). All three bacteriophages were stable at pH 4–10 (FKP3, Kruskal-Wallis *p* = 0.849; FKP10, ANOVA *p* = 0.700 and FKP14, Kruskal-Wallis *p* = 0.957), whereas they lost their stability at pH 2 and 12.

All three bacteriophages were stable at temperatures between 4°C and 50°C. The bacteriophage FKP3 reduced 3.9 Log when exposed to a temperature of 70°C (*p* = 4.22 × 10^−11^***), and FKP10 decreased 2.8 Log at 60°C (*p* = 1.0) and 6.6 Log at 70°C (*p* = 0.258) (Figure 6A). Regarding pH stability, similar behavior was observed in the three bacteriophages, which maintained stability at a pH between 4 and 10 (FKP3, *p* = 0.849; FKP10, *p* = 0.700; FKP14, *p* = 0.957); however, they were susceptible to extreme pH levels of 2 and 12, so a complete reduction in bacteriophage titers was observed for both pHs (Figure 6B).

#### 3.2.7 Transmission electron microscopy

**Figure 7:**
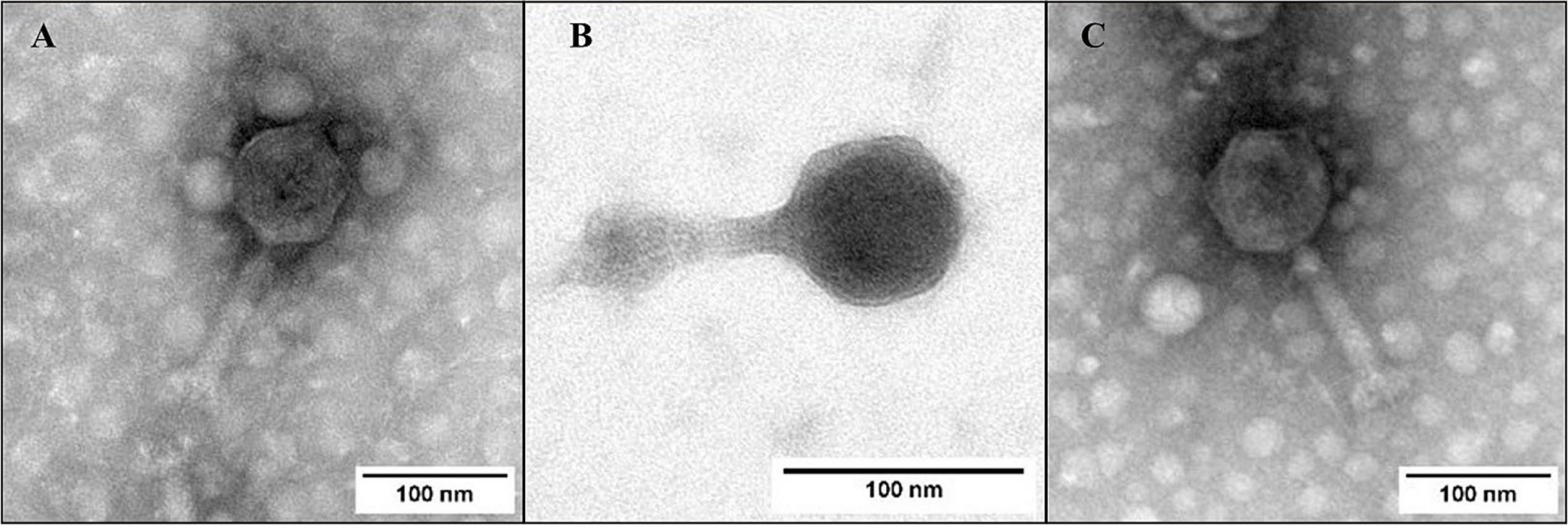
Bacteriophage morphology. **(A–C)** Show photographs taken by transmission electron microscopy (TEM) of FKP3, FKP10, and FKP14, respectively. The three bacteriophages had tails sized 165.6 nm ±1.2 and 208.1 nm ±1.1. The scale bar represents 100 nm.

Phages FKP3, FKP10, and FKP14 had approximate lengths of 206.2 ± 3.6, 165.6 ± 1.2 and 208.1 ± 1.1 nanometers, respectively, and all of them had tail, so they belong to the *Caudoviricetes* class. All three phages had icosahedral isometric capsids with sizes of 89.05 ± 2.6 nm (FKP3), 73.8 ± 3 nm (FKP10) and 92.2 ± 1.9 nm (FKP14). The tails were straight and short with approximate sizes of 116.7 ± 0,5 nm (FKP3), 89.09 ± 1,6 nm (FKP10) and 114.2 ± 2.4 nm (FKP14) (Figure 7).

#### 3.2.8 Genome characteristics and annotation

The genome features are described in Table 2. Bacteriophages had double-stranded DNA (dsDNA), and the three presented high-quality genomes with integrity >99%. The genomes had a length between ~142 and ~ 159 kbp; further, the FKP3 (GenBank: PP895363.1) genome was linear, whereas FKP10 (GenBank: PP974338.1) and FKP14 (GenBank: PP974339.1) had circularly permuted genomes. More than 230 open reading frames (ORFs) and coding sequences (CDS) were annotated; but, most corresponded to genes encoding hypothetical proteins (~60%) and about 33% corresponded to genes encoding proteins with known functions. These genes were classified into functional categories, such as structural and packaging proteins, associated with lysis, moron, integration and excision, auxiliary metabolic genes and host takeover, and DNA, RNA, and metabolism genes (Figure 6). All three phages had CDS associated with lytic lifestyles such as Rz-like spanins, endolysin, lysis inhibitors, and endolysins; however, FKP3 also had a CDS (0063) that was identified as a site-specific recombination directionality factor (RDF) and assigned to the integration and excision module. The presence of this recombination factor decreased the probability to 46.25% that this bacteriophage was virulent when implementing the BACHLIP tool; however, both the Phage AI and phaTYP tools predicted with high probabilities (> 90%) that all three bacteriophages were virulent (Table 2). No virulence-associated or antimicrobial resistance genes were detected in the bacteriophages.

**Table 2:** Genomic profile of FKP3, FKP10, and FKP14 bacteriophages active against *K. pneumoniae*.

Genomic profile	*Klebsiella* phage FKP3	*Klebsiella* phage FKP10	*Klebsiella* phage FKP14
Size (bp)	142277	159358	157205
Genome organization	Linear	Circularly permuted	Circularly permuted
Depth	125X	111X	139X
GC content (%)	39.56	46.57	46.3
Completeness	100	100	99.94
ORF	305	246	233
CDS	303	244	231
Hypothetical proteins	212	140	129
Functional proteins	91	104	102
tRNA*	21	8	7
Termini	Short direct terminal repeats (DTRs) of 317 bp length	Headful packaging (PAC)	Non identified**
Taxonomy			
Family	*Stephanstirmvirinae*	*Ackermannviridae*	*Ackermannviridae*
Genus	*Justusliebigvirus*	*Taipeivirus*	*Taipeivirus*
*In silico* replicative cycle			
PhageAI (virulent)	91.74%	92.49%	92.80%
BACHLIP (virulent)	46.25%	98.39%	94.50%
PhaTYP (virulent)	1.0	1.0	1.0

GC, guanine-cytokine content; ORF, Open Reading Framework; CDS, coding sequences; tRNA, transfer RNA. * Four transfer RNAs were identified in all phages (tRNAMet, tRNATyr, tRNAAsn, tRNAGln). ** Owing to the lack of identified termini in FKP14 genome, the genome was re-arranged it using the large terminase subunit as the start base through the Pharokka pipeline, and as recommended in Shen and Millard 2021.

#### 3.2.9 Comparative genomics and phylogenetic analysis

**Figure 8:**
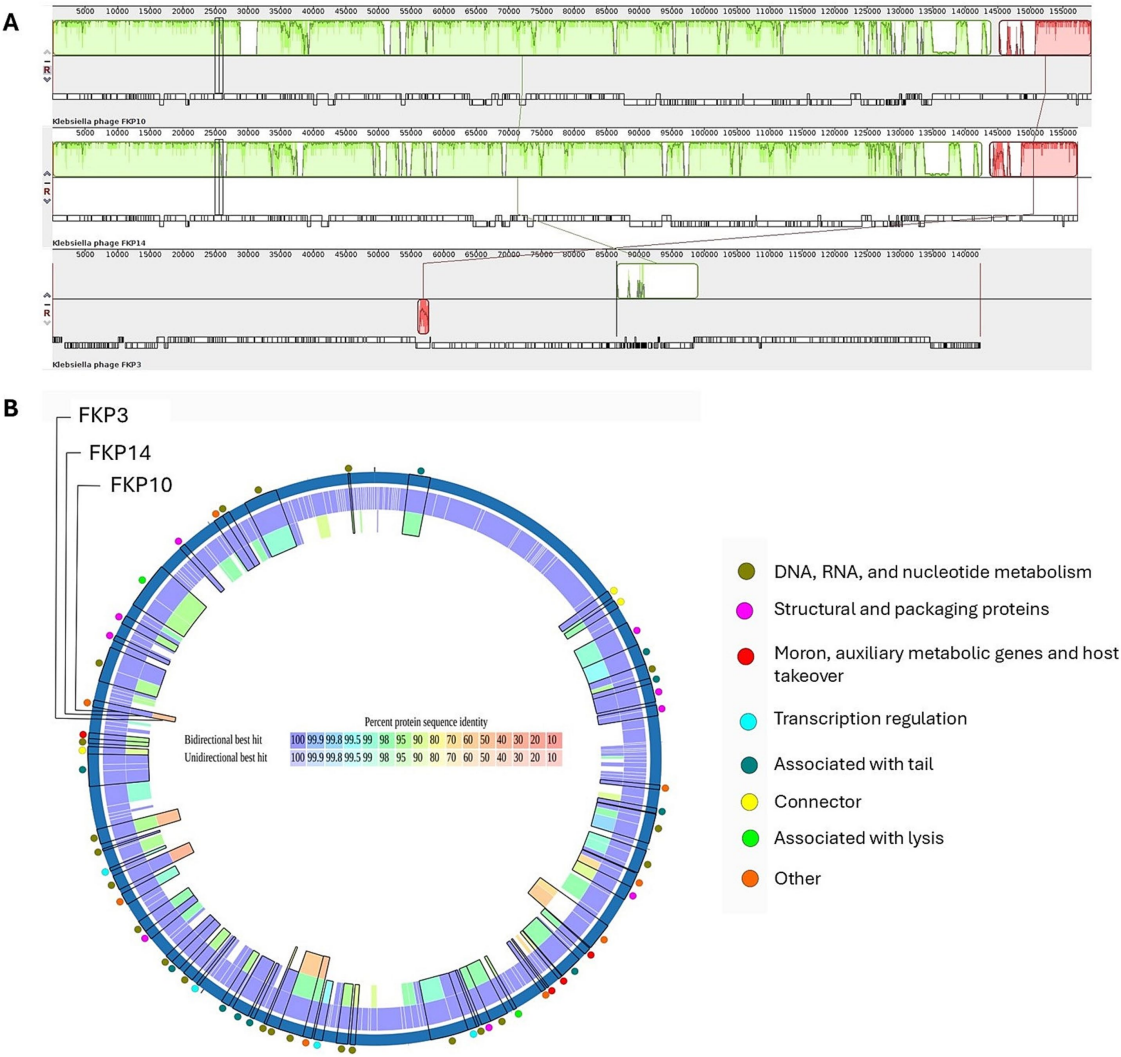
Comparative genomics of *K. pneumoniae* bacteriophages. **(A)** Presents a comparison of the genomes of FKP10, FKP14, and FKP3 phages generated using Progressive MAUVE. These phage genomes are arranged horizontally with homologous genomic regions (locally collinear blocks) delineated in the same color. **(B)** Presents a comparative circular map of the protein-coding genes of FKP3, FKP10, and FKP14 phages using PATRIC. The outer colored circles indicate the putative functions of the protein-coding genes. Proteomic analysis revealed the orthology of protein-coding genes between FKP10 and FKP14 phages with >90% sequence identity **(B)**. These protein-coding genes were linked to head and packaging, moron, auxiliary metabolic functions, host interaction, transcription regulation, tail, and lysis. Notably, three lysis-associated genes: endolysin (FKP10 ORF 3, FKP14 ORF 61), RIIB (FKP10 ORF 199, FKP14 ORF 172), and RIIA lysis inhibitors (FKP10 ORF 200, FKP14 ORF 173), had >90% protein sequence identity and were shared exclusively by FKP10 and FKP14. Phage FKP3 exhibited lower protein sequence identity (up to 50%) than FKP10 and FKP14 phages. The shared protein-coding genes among the three phages were involved in DNA, RNA, and nucleotide metabolism.

**Figure 9:**
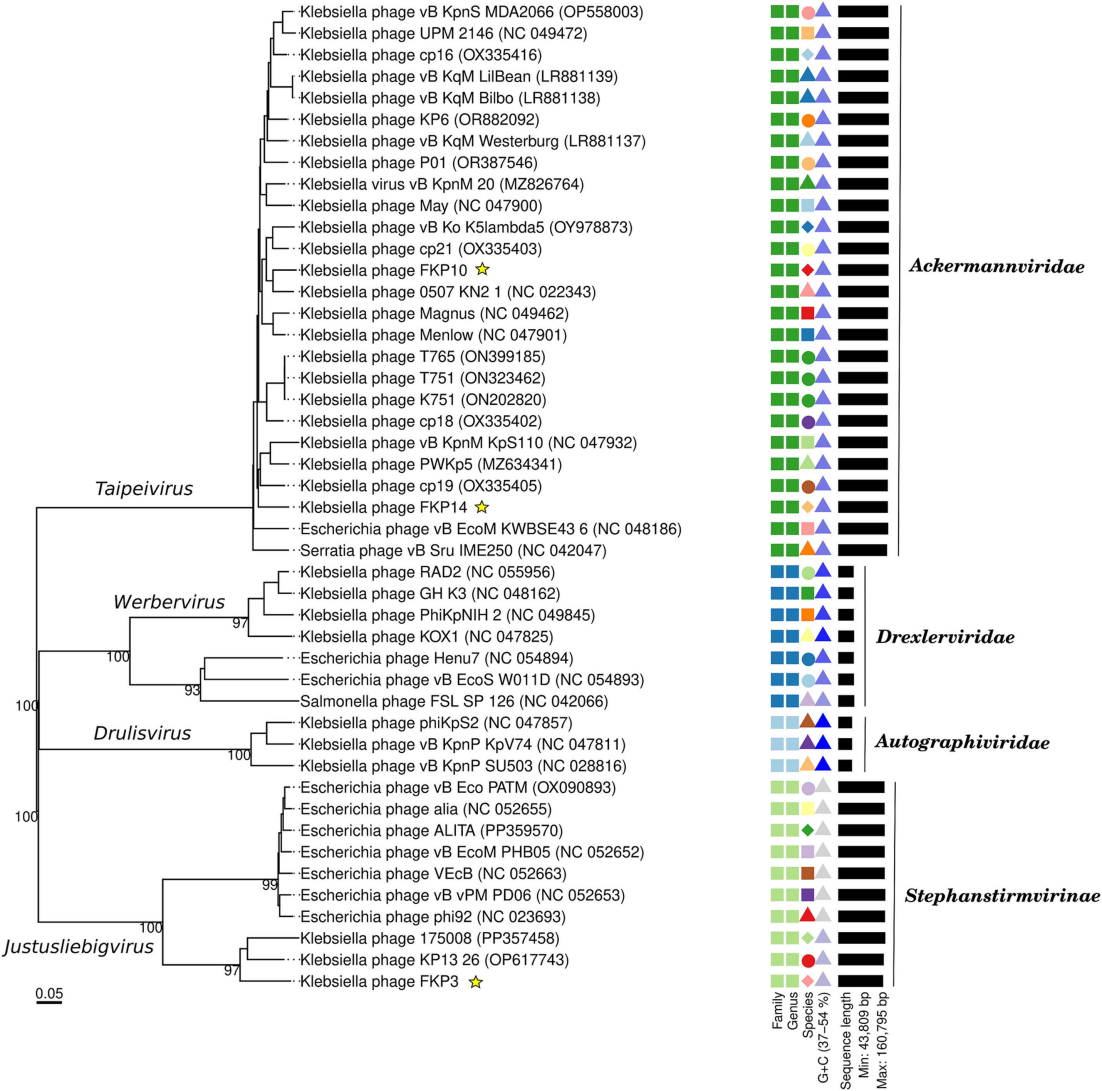
Genome-based phylogenetic tree of bacteriophages FKP3, FKP10 and FKP14. The phylogenetic tree was generated by VICTOR nucleotide pairwise comparisons. Yellow stars indicate the phages of interest in this study. The colors and shapes on the right indicate similarities of bacteriophages according to characteristics such as family, genus, species, guanine-cytosine content, and genome size.

Figure 8A shows multiple alignments of *Klebsiella* phage genomes containing two syntenic colinear blocks (homologous regions). This alignment was consistent with high ANIb values between FKP10 and FKP14 (ANIb% between 95.83 to 96.04%), indicating greater genomic similarity, and lower ANIb values for FKP3 (ANIb% with FKP10 between 62.08 to 62.26%; ANIb% with FKP14 between 67.11 to 70.08%). Based on these results, FKP10 and FKP14 phages were more closely related to each other than to FKP3. Figure 8B presents a comparative circular map of the protein-coding genes of the three annotated genomes. Furthermore, a genome-to-genome distance-based phylogenetic tree was constructed using closely related phages based on ANIb values and Viptree Sg scores, with distant members set as outgroups from the *Drexlerviridae* and *Autographiviridae* families. This phylogenetic tree was composed of 4 families, 4 genera and 43 species clusters; and the phages FKP10 and FKP14 belonged to the *Ackermannviridae* family and *Taipeivirus* genus, whereas FKP3 belonged to the S*tephanstirmvirinae* and *Justusliebigvirus* families (Figure 9).

## 4 Discussion

Although bacteriophages were discovered more than 100 years ago, during the last two decades, the use of these viruses to combat antibiotic-resistant bacteria has increased, making them attractive alternatives for the control of carbapenem-resistant clones of *K. pneumoniae* (CRKP), which have successfully spread in clinical and environmental settings. This study allowed the isolation of highly specific bacteriophages against the CRKP strains CG258 and ST307 with promising biological, structural, and genomic traits.

Although it is important to isolate specific bacteriophages against the strains of interest, it is necessary to perform an individual characterization of bacteriophages to determine their safety and predict their performance in various applications, whether in biocontrol or phage therapy. At present, there are no standardized guidelines that determine which characteristics should be evaluated for the subsequent implementation of bacteriophages; however, some entities, such as the Food and Drug Administration (FDA) and the European Medicines Agency (EMA), have made recommendations to ensure the use of bacteriophages in various applications. These recommendations include confirmation of activity against the strains of interest, origin of the phage, effective concentration of the phage, absence of antibiotic resistance genes, virulence genes, and genes associated with integration or lysogeny (Yang et al., 2023; Furfaro et al., 2018). However, other important characteristics could help predict the performance of bacteriophages against different isolates, including host range, plating efficiency, and infection or killing curves. Considering that these methodologies can be diverse, the selection of criteria for characterization must be aligned with the objective of the research and the type of application.

In recent years, it has become more common to find publications that focus on identifying bacteriophages against high-risk clones; some studies have described the isolation of active phages against clones such as ST11, ST101, ST16, ST13, and ST15, among others (Martins et al., 2022a; Fang and Zong, 2022; Tan et al., 2019; Ciacci et al., 2018; Laforêt et al., 2022; Horváth et al., 2020). Likewise, the isolation of active bacteriophages against CRKP-ST258 has been reported, whereas publications of active phages against emerging clones such as ST307 are limited (Tisalema-Guanopatín et al., 2023; Thiry et al., 2019; D’Andrea et al., 2017; Venturini et al., 2020; Hesse et al., 2020; Ponsecchi et al., 2024).

The FKP3, FKP4, and FKP14 phages exhibited a broad host range with high specificity against CG258 isolates (56 to 60%). This finding aligns with a study by D’Andrea et al., where *K. pneumoniae* bacteriophages displayed a host range covering 52.4% of strains from CG258 (clade II) (D’Andrea et al., 2017). On the contrary, a study published in 2020 by Venturini C et al. reported the isolation of bacteriophages from ST258 (CG258) strains, which only had activity against their host bacteria and not against other strains tested (Venturini et al., 2020).

The high specificity of infection observed in the selected bacteriophages could be associated with the presence of common bacterial receptors in these clones, which are specifically recognized by the receptor binding proteins (RBP) of the phage (De Jonge et al., 2018). In *K. pneumoniae* phages have been described important receptors involved in the specificity of infection, such as capsular type, in these sense Venturini et al. isolated specific bacteriophages against *K. pneumoniae* from CG258, finding a correlation between the host range of the bacteriophages and the capsular type, as well as with the clade to which these clones belonged (Venturini et al., 2020).

On the other hand, phages FKP10 and FKP12 show a wide host range of 66.7–93.3% against CRKP from ST307; which is in line with previous studies conducted at Lyon University Hospital in France (2021), where it was described the isolation and characterization of one *K. pneumoniae* jumbo phage with activity against 80% (24/30) ST307 clones (unpublished data). Recent studies reported the isolation of two phages active against CRKP-ST307 with a host range of up to 85.8% (10/12) of isolates (Ponsecchi et al., 2024).

Finally, phage studies against CRKP from CG258 and ST307 have not only focused on characterization but also evaluated other aspects of the phage’s activity related to the mechanisms of resistance to bacteriophages and the effects of bacterial fitness during this process (Thiry et al., 2019; Hesse et al., 2020; Hesse et al., 2021; De Angelis et al., 2021).

The findings of this study are of great interest considering that few publications have reported the isolation and characterization of CRKP phages belonging to CG258 and ST307. Moreover, not all studies that report the isolation of phages against these clones perform host range using a large number of isolates, and in some cases, authors did not evaluate the host range (Tisalema-Guanopatín et al., 2023; D’Andrea et al., 2017; Venturini et al., 2020). Furthermore, many of these studies only assessed host range using the spot test and did not confirm productive phage infection by determining plaque formation in susceptible strains, which may have overestimated phage activity and host range results (D’Andrea et al., 2017; Venturini et al., 2020). Unlike the limitations of these studies, in this work, the productive infection of bacteriophage was determined, and we confirmed that the results of the spot test effectively overestimated the host range from 2.4 to 29.4%, in comparison with the host range assessed by the determination of plaque formation, highlighting the importance of this confirmation. Finally, our findings showed that bacteriophages efficiently infected 94% of the susceptible strains in the host range (EOP values ≥0.5); which again underlines the importance of this study in comparison to others reported, where the infection efficiency of phages against these important clones is not evaluated (Tisalema-Guanopatín et al., 2023; D’Andrea et al., 2017). Overall, the EOP results for non-CG258 and non-ST307 CRKP phages are diverse and depend on each bacteriophage. Some studies have reported high infection efficiency in the 54.5% of the strains (Kim et al., 2023; Balcão et al., 2022).

Evaluation of the host range in many strains and determination of EOP are important procedures for characterizing bacteriophages because they allow estimation of the probability of success of a bacteriophage against specific groups of bacteria during its application. The host range allows us to identify which bacteria are susceptible to the action of a phage by recognizing common receptors between them. Further, EOP shows how bacteria are permissive for phage replication, which allows us to determine how well phages replicate in infected bacteria compared to their host bacteria by identifying productive infection (Khan Mirzaei and Nilsson, 2015; Kutter, 2009). Permissiveness is also related to the presence of resistance mechanisms to bacteriophages; therefore, our EOP results with values equal to or greater than 1 probably indicate that the strains do not share phage resistance mechanisms; thus, they replicate efficiently in most strains (Hyman and Abedon, 2010).

Although individual bacteriophages showed high species specificity (100%) and specific activity against CRKP from CG258 and ST307, the use of bacteriophage cocktails increased the intra-species host range up to 85.7% (*n* = 36/42) of isolates belonging to both clones, which makes it a very interesting alternative for its implementation in places where both clones circulate frequently, such as hospital settings. The activity of the phages of this study against both clones (85.7%) could indicate additive activity of the phages FKP3, FKP14 (CG258), and FKP10 (ST307); however, it was observed that the cocktail increased the host range up to 80.8% of the CG258 strains and 93.8% of the ST307 strains vs. 56 and 66.7% of the activity obtained with the individual phages, respectively. Furthermore, some bacteria that were not susceptible to either phage individually were susceptible to the cocktail, indicating the possible synergistic activity of the phages in these strains. This work also allowed us to determine whether the cocktail’s bacteriophages were replicating during infection by confirming the formation of plaques in susceptible strains, an aspect that has been rarely evaluated in other studies. These findings are of great relevance because they reject the effect of “lysis from without” and confirm that a productive infection of the cocktail phages is taking place; besides, demonstrate that antagonism phenomena during co-infection probably do not occur in most strains (Molina et al., 2022; Kerr et al., 2008).

Several studies have described the use of bacteriophage cocktails to control *K. pneumoniae*; however, a specific cocktail has not been described to control high-risk clones such as CG258 and ST07. Other publications have focused on the evaluation of phage cocktails in biofilm-producing *K. pneumoniae* or animal infection models to determine the activity of the cocktails against specific strains (Zurabov et al., 2023; Kelly and Jameson, 2024; Liang et al., 2023; Singh et al., 2022). Furthermore, most studies that evaluate the activity of cocktails only determine their effectiveness through a killing curve with some strains, and there are few publications that re-evaluate the performance of cocktails with various isolates as is usual with the host range (Jokar et al., 2023). According to a study published in 2023, the use of a cocktail of 4 bacteriophages increased the host range from 52 to 75% of the isolates, preventing bacterial regrowth (Jokar et al., 2023). Another study published in Kenya determined the performance of a cocktail in 8 strains and reported no differences between the administration of individual or combination bacteriophages (Michodigni et al., 2022). These studies contrast with the present work, in which various cocktails were evaluated against several isolates and productive infection was confirmed. Furthermore, the use of cocktails significantly increased the host range against CG258 and ST307, with higher percentages observed than those reported in previous studies, indicating a greater probability of cocktail success.

Additionally, the performance of the cocktails was determined using the infection curve obtained after 23 h, which differs from some studies on *K. pneumoniae* phages in which evaluation is shorter, making it more difficult to identify phage-resistant subpopulations over time (Fayez et al., 2023). In general, our phages showed significant control of bacterial growth but did not exhibit complete elimination. Furthermore, the cocktails presented mixed results, and in some cases, an improvement in the performance of the cocktail was observed at low MOIs compared to the performance of the individual phage. The use of the cocktail at MOIs of 0.1 and 0.01 on the host bacteria of phages FKP3 (CRKP-ST 512, strain KP58) and FKP10 (CRKP-ST307, strain KH45) delayed the appearance of phage-resistant bacteria; compared to the individual phage results at the same MOIs. Therefore, the cocktail achieved a performance like the individual phage FKP3 and FKP10 at an MOI of 1 (FKP3, 82.70% control first 6 h; FKP10, 67.55% control first 12 h), whose concentration was the best in all cases. These findings coincide with those of other studies in which the use of the cocktail delayed the appearance of subpopulations resistant to phages (Kondo et al., 2023). However, other publications have reported that the use of cocktails did not perform significantly better than that of phage alone (Concha-Eloko et al., 2023); which was also evidenced in our results with the host bacteria of phage FKP14 (CRKP-ST258, strain KR06). These findings demonstrate the importance of evaluating cocktails using infection curves with different strains, considering that the behavior may vary and could be related to the resistance mechanisms of bacteria to phages.

It is well known that the use of cocktails offers advantages compared to the administration of individual phages because they expand the host range, and their use decreases the probability of selecting phage-resistant bacteria. In this sense, it is ideal to use combinations of phages with affinity for different receptors, considering that the use of bacteriophages that recognize the same receptor could be ineffective due to the development of bacterial resistance mechanisms related to mutations that cause cross resistance (Abedon et al., 2021; Yoo et al., 2024). The use of phages that recognize different receptors can delay or prevent the emergence of resistance; which was observed in this study; However, in our case, it could be necessary to use an additional phage to control the remaining resistant subpopulations, administer the phages from the cocktail sequentially over time, or use combinations of bacteriophages with other compounds. Some studies have documented a greater reduction in bacterial growth when *K. pneumoniae* phage cocktails are combined with antibiotics such as meropenem and tigecycline (Martins et al., 2022b; Michodigni et al., 2022). Furthermore, it has been reported that the use of bacteriophages and chemical disinfectants is more effective in eliminating biofilms and bacteria on surfaces (Chen et al., 2024).

On the other hand, the results of the one-step curve indicated rapid replication of the bacteriophages and rapid release of viral particles (latency time 10 min). Some studies have shown that phages with short latency periods lyse more bacteria at each time, demonstrating their potential for rapid control of bacterial populations (Fang et al., 2023). The burst size results were obtained when the infection was carried out at an MOI of 0.01 and under these conditions there was a low production of viral progeny (5–18 per infected cell). In this sense, the burst size results coincide with those observed in the elimination curve at an MOI of 0.01, where there was no evident control of bacterial growth, which is related to the low production of bacteriophages that infect adjacent bacteria. Although it is considered that the bacteriophages with the greatest potential are those with a large burst size, it has been reported that bacteriophages with small burst sizes are associated with short lysis cycles, as observed in this study. This can also be considered an advantage by favoring the development of several replication cycles in shorter timeframes and faster viral particle production (Shao and Wang, 2008). Other authors have described small burst sizes (6 to 63 bacteriophages per infected cell) in *K. pneumoniae* phages, however, the possible limitations caused by burst size could be solved with the use of higher MOI or the combined use of other bacteriophages, antibiotics, or compounds (Tisalema-Guanopatín et al., 2023; Peng et al., 2023; Gordillo Altamirano and Barr, 2019).

Finally, the bacteriophages that made up the best cocktail belonged to the class of *Caudoviricetes* and to the families *Stephanstirmvirinae* (FKP3) and *Ackermannviridae* (FKP10 and FKP14), with the latter being one of the most frequently reported phages against *Klebsiella* (Tisalema-Guanopatín et al., 2023; Assafiri et al., 2021). All bacteriophages were virulent; however, in phage FKP3, a gene was identified that encodes an RDF (recombination directionality factor) protein. This gene is involved in the directionality of site-specific recombination mediated by integrases (Lewis and Hatfull, 2001). However, FKP3 did not possess other genes related to the lysogenic cycle, and tools *in silico* indicated a high probability (91.74%) of being virulent. Some authors have reported that RDF proteins can accomplish functions related to the process of DNA replication; therefore, the acquisition of the RDF gene may be due to evolutionary processes and requires further studies of its transcription and functionality (Payaslian et al., 2021). In addition, no genes encoding virulence or antibiotic resistance factors were found in the three genomes, which supports the safety of these bacteriophages for their implementation in future applications. Other results showed that bacteriophages harbor several tRNAs in their genomes, which are associated with lytic replication cycles (Bailly-Bechet et al., 2007; Nepal et al., 2022). Likewise, the presence of tRNA enables viral proteins to be translated more efficiently, reducing latency times, which could be evidenced in the three bacteriophages evaluated, whose eclipse and latency periods were short (Bailly-Bechet et al., 2007). Finally, genes associated with lysis proteins, such as endolysins and Rz-type spins, were identified in the genomes of the three bacteriophages, and these genes are related to Gram-negative lysis processes (Kongari et al., 2018; Briers et al., 2014).

The evaluation of the biological and structural characteristics of the bacteriophages isolated in this study supports the development of new applications, particularly in critical scenarios with high CRKP levels in CG258 and ST307. These bacteriophages could be used at higher MOIs in scenarios where the target bacterial population is not very high and under conditions where the bacteria have slower replication rates that allow control of the bacterial population. In this context, bacteriophages could be used as part of a surface disinfection strategy in hospital settings given that CRKPs CG258 and ST307 are frequently found in these settings and spread easily, causing healthcare-associated infections (Centeleghe et al., 2023). In addition, its use could be explored in hospitals and community wastewater where these pathogens have been frequently reported (Jassim et al., 2016). Finally, considering the specificity of infection against these high-risk clones, the bacteriophages isolated in this study could be implemented as diagnostic and epidemiological surveillance tools, considering that CRKP of CG258 and ST307 are the main circulating clones at the local and global levels (Fu et al., 2015).

The limitations and perspectives of this study are related with the evaluation of the mechanism of action of the bacteriophages and receptors involved. Furthermore, the evaluation of phage-resistant populations, resistance mechanisms, and phenomena such as trade-off (resensitization to antibiotics, altered metabolism, decreased virulence) (Burmeister et al., 2020; Gordillo Altamirano et al., 2021; Chan et al., 2016; Fujiki et al., 2023). Finally, additional combinations with antibiotics or other compounds and the study of the functionality of some genes of biotechnological interest, such as endolysins, are expected.

## 5 Conclusion

In this study, cocktails of bacteriophages with high activity against CRKP isolates belonging to the successful clones CG258 and ST307 were obtained. Through the individual characterization of each of these bacteriophages, promising biological, structural, and genomic traits were identified, infection specificity against *K. pneumonie* of CG258 and ST307, high lytic activity, short latency periods, rapid replication cycles, stability at varying pH and temperature conditions, and the absence of genes associated with antibiotic resistance and virulence. Together, these results show the potential of these bacteriophage combinations for the control of carbapenem-resistant *K. pneumoniae* of the CG258 and ST307 and, in turn, constitute a starting point for future *in vitro* and *in vivo* studies where these bacteriophages are implemented in clinical and environmental scenarios. The characterization results obtained in this work allow the prediction of the performance of bacteriophages during applications.

## Data Availability

The datasets presented in this study can be found in online repositories. The names of the repository/repositories and accession number(s) can be found below: https://www.ncbi.nlm.nih.gov/ (PP895363.1, PP974338.1, and PP974339.1).

## Footnotes

^1^ https://sourceforge.net/projects/bbmap/

^2^ https://blast.ncbi.nlm.nih.gov/Blast.cgi

^3^ https://github.com/gbouras13/phold
