# Supplementary material for: High activity and specificity of bacteriophage cocktails against carbapenem-resistant Klebsiella pneumoniae belonging to the high-risk clones CG258 and ST307
Source: Front Microbiol. 2024 Dec 9;15:1502593. doi: 10.3389/fmicb.2024.1502593 (PMC11663894; doi:10.3389/fmicb.2024.1502593)
Supplement: Supplementary file 1 [file Data_Sheet_1.pdf]

Supplementary Material

Supplementary Table S1. Characteristics of *K. pneumoniae* host isolates used for the search and isolation of bacteriophages.

Host bacteria	ID	Source	Carbapenemase	Clonal group (CG)	Sequence type (ST)	Other antibiotics resistance			
						Aminoglycosides	Fluoroquinolones	Glycylcycline	Nitrofurans
***Klebsiella pneumoniae* (n=3)**	KL15	Hospitalized patients	*bla*-$_{KPC-3}$	258	512	Resistant	Resistant	Resistant	ND
	KL56		*bla*-$_{KPC-2}$	258	258	Resistant	Resistant	Resistant	ND
	KH18		*bla*-$_{KPC-2}$	307	307	Resistant	Resistant	ND	Resistant

ND, No data. All bacterial isolates are part of the strain collection of the Grupo de Investigación en Microbiología Básica y Aplicada (MICROBA) and were collected in the city of Medellin, Colombia. Identification and susceptibility tests were performed using the semi-automated method VITEK® 2, genes that encoded carbapenemases were identified by PCR and sequenced to determine gene-variants. Finally, molecular typing was performed using Multilocus sequence typing (MLST).
