# Supplementary material for: High activity and specificity of bacteriophage cocktails against carbapenem-resistant Klebsiella pneumoniae belonging to the high-risk clones CG258 and ST307
Source: Front Microbiol. 2024 Dec 9;15:1502593. doi: 10.3389/fmicb.2024.1502593 (PMC11663894; doi:10.3389/fmicb.2024.1502593)
Supplement: Supplementary file 2 [file Data_Sheet_2.pdf]

Supplementary Material

Supplementary Table S2. Bacterial isolates used for the evaluation of the intra-species host range of bacteriophages active against *K. pneumoniae*.

Bacteria	Characteristic	Clonal group (CG) or Clonal complex (CC)	Sequence type (ST)	Number of isolates (n=100)
Klebsiella pneumoniae	Carbapenems resistant, *bla-KPC+*	CG258	ST512 (n = 4)	25
			ST258 (n = 20)	
			ST11 (n = 1)	
		CC307	ST307	15
		CC14	ST14	10
		Other	ST17, ST23, ST40, ST45, ST129, ST140, ST151, ST231, ST259, ST268, ST444, ST526, ST560, ST971, ST1198, ST1377, ST1533, ST1661, ST1703, ST1704, ST1705, ST1706, ST1707, ST1708, ST1887	25
	Carbapenems susceptible	ND	ND	25

ND, No data. All bacterial isolates are part of the strain collection of the Grupo de Investigación en Microbiología Básica y Aplicada (MICROBA) and were collected in the city of Medellin, Colombia. Identification and susceptibility tests were performed using the semi-automated method VITEK® 2, genes that encoded carbapenemases were identified by PCR and molecular typing was performed using Multilocus sequence typing (MLST).
