# Supplementary material for: High activity and specificity of bacteriophage cocktails against carbapenem-resistant Klebsiella pneumoniae belonging to the high-risk clones CG258 and ST307
Source: Front Microbiol. 2024 Dec 9;15:1502593. doi: 10.3389/fmicb.2024.1502593 (PMC11663894; doi:10.3389/fmicb.2024.1502593)
Supplement: Supplementary file 3 [file Data_Sheet_3.pdf]

Supplementary Material

Supplementary Table S3. Antibiotic resistance of the bacterial isolates used for the evaluation of the intra-species host range of bacteriophages active against *K. pneumoniae.*

Bacteria	Clonal Group or Clonal complex	Sequence Type	Carbapenemase	Amikacin	Gentamicin	Nalidixic acid	Ciprofloxacin	Nitrofurantoin	Tigecycline	TMP/SMX
KL045	258	512	KPC-3	64 (R)	8 (R)	ND	4 (R)	ND	4 (R)	ND
KL004	258	512	KPC-3	64 (R)	4 (S)	32 (R)	4 (R)	512 (R)	ND	320 (R)
KL015	258	512	KPC-3	64 (R)	4 (S)	32 (R)	4 (R)	ND	2 (R)	ND
KP058	258	512	KPC-3	64 (R)	4 (S)	ND	4 (R)	ND	2 (R)	ND
KL009	258	512	KPC-2	16 (S)	1 (S)	32 (R)	4 (R)	ND	2 (R)	ND
KH001	258	258	KPC-3	64 (R)	1 (S)	32 (R)	4 (R)	512 (R)	ND	20 (R)
KH004	258	258	KPC-3	16 (S)	16 (R)	32 (R)	4 (R)	ND	2 (R)	ND
KH005	258	258	KPC-3	16 (S)	16 (R)	32 (R)	4 (R)	ND	4 (R)	ND
KH013	258	258	KPC-3	64 (R)	4 (S)	ND	4 (R)	ND	2 (R)	ND
KH028	258	258	KPC-3	64 (R)	4 (S)	ND	4 (R)	ND	2 (R)	ND
KH036	258	258	KPC-3	64 (R)	16 (R)	ND	4 (R)	ND	2 (R)	ND
KL008	258	258	KPC-3	64 (R)	4 (S)	32 (R)	4 (R)	ND	2 (R)	ND
KL055	258	258	KPC-3	4 (S)	16 (R)	ND	4 (R)	ND	0.5 (S)	ND
KL011	258	258	KPC-3	64 (R)	4 (S)	32 (R)	4 (R)	ND	2 (R)	ND
KL013	258	258	KPC-3	64 (R)	4 (S)	32 (R)	4 (R)	ND	2 (R)	ND
RK015	258	258	KPC-3	64 (R)	4 (S)	ND	4 (R)	ND	2 (R)	ND
KL054	258	258	KPC-2	16 (S)	16 (R)	ND	4 (R)	ND	8 (R)	ND
KL056	258	258	KPC-2	64 (R)	8 (R)	ND	4 (R)	ND	4 (R)	ND

KP027	258	258	KPC-2	16 (S)	16 (R)	32 (R)	2 (R)	ND	4 (R)	ND
KP029	258	258	KPC-2	16 (S)	16 (R)	32 (R)	1 (S)	64 (R)	ND	320 (R)
KP042	258	258	KPC-2	4 (S)	1 (S)	ND	0.25 (S)	ND	0.5 (S)	ND
KR006	258	258	KPC-2	64 (R)	8 (R)	ND	4 (R)	ND	ND	ND
KR015	258	258	KPC-2	4 (S)	16 (R)	ND	4 (R)	512 (R)	ND	320 (R)
RK052	258	258	KPC-2	64 (R)	4 (S)	ND	4 (R)	ND	ND	ND
KP031	258	11	KPC-2	2 (S)	16 (R)	ND	4 (R)	ND	2 (R)	ND
KL024	258	258	KPC-3	2 (S)	1 (S)	ND	4 (R)	ND	4 (R)	ND
KH018	307	307	KPC-2	8 (S)	16 (R)	ND	4 (R)	64 (R)	ND	40 (R)
KH021	307	307	KPC-2	8 (S)	16 (R)	ND	4 (R)	ND	0.5 (S)	ND
KH033	307	307	KPC-2	2 (S)	16 (R)	ND	4 (R)	ND	8 (R)	ND
KH041	307	307	KPC-2	2 (S)	16 (R)	ND	4 (R)	ND	2 (R)	ND
KH042	307	307	KPC-2	16 (S)	16 (R)	ND	4 (R)	ND	4 (R)	ND
KH043	307	307	KPC-2	2 (S)	16 (R)	ND	4 (R)	ND	4 (R)	ND
KL049	307	307	KPC-2	2 (S)	16 (R)	ND	4 (R)	ND	1 (S)	ND
KH016	307	307	KPC-3	8 (S)	16 (R)	ND	4 (R)	ND	2 (R)	ND
KH020	307	307	KPC-3	2 (S)	16 (R)	ND	4 (R)	ND	ND	ND
KH023	307	307	KPC-3	2 (S)	16 (R)	ND	4 (R)	ND	1 (S)	ND
KH035	307	307	KPC-3	2 (S)	16 (R)	ND	4 (R)	ND	2 (R)	ND
KH037	307	307	KPC-3	2 (S)	16 (R)	ND	4 (R)	ND	2 (R)	ND
KL057	307	307	KPC-3	64 (R)	16 (R)	ND	4 (R)	ND	1 (S)	ND
KH022	307	307	KPC-2	2 (S)	16 (R)	ND	0.25 (S)	ND	0.5 (S)	ND
KH045	307	307	KPC-2	2 (S)	1 (S)	ND	4 (R)	ND	ND	ND
KL044	307	307	KPC-2	4 (S)	16 (R)	ND	4 (R)	ND	2 (R)	ND
KC001	ND	14	KPC-2	16 (S)	16 (R)	32 (R)	2 (R)	ND	ND	ND
KH002	ND	14	KPC-2	2 (S)	1 (S)	32 (R)	4 (R)	ND	4 (R)	ND
KP010	ND	14	KPC-2	64 (R)	ND	32 (R)	2 (R)	ND	2 (R)	ND
KP024	14	14	KPC-2	16 (S)	16 (R)	32 (R)	1 (S)	ND	4 (R)	ND
KP030	ND	14	KPC-2	16 (S)	16 (R)	32 (R)	2 (R)	128 (R)	ND	320 (R)
KP036	ND	14	KPC-2	16 (S)	16 (R)	ND	4 (R)	ND	8 (R)	ND
KP043	ND	14	KPC-2	16 (S)	16 (R)	ND	2 (R)	ND	4 (R)	ND

KP050	ND	14	KPC-2	16 (S)	16 (R)	ND	1 (S)	ND	4 (R)	ND
KR007	ND	14	KPC-2	16 (S)	16 (R)	ND	1 (S)	128 (R)	ND	320 (R)
KH010	ND	14	KPC-3	2 (S)	1 (S)	16 (S)	1 (S)	ND	1 (S)	ND
KH011	ND	17	KPC-2	2 (S)	1 (S)	4 (S)	0.25 (S)	ND	0.5 (S)	ND
KH038	ND	23	KPC-2	2 (S)	1 (S)	ND	0.25 (S)	ND	0.5 (S)	ND
KP034	ND	40	KPC-3	16 (S)	1 (S)	ND	4 (R)	ND	2 (R)	ND
KH003	ND	45	KPC-3	2 (S)	1 (S)	32 (R)	2 (R)	512 (R)	ND	20 (R)
KH008	ND	129	KPC-2	4 (S)	1 (S)	32 (R)	1 (S)	ND	8 (R)	ND
KC008	ND	140	KPC-2	2 (S)	1 (S)	ND	0.25 (S)	ND	1 (S)	ND
KH014	ND	151	KPC-3	16 (S)	16 (R)	ND	2 (R)	ND	2 (R)	ND
KL037	ND	231	KPC-3	64 (R)	4 (S)	ND	4 (R)	ND	4 (R)	ND
KL036	ND	259	KPC-2	2 (S)	16 (R)	ND	0.5 (S)	ND	1 (S)	ND
KH007	ND	268	KPC-3	2 (S)	16 (R)	16 (S)	1 (S)	ND	2 (R)	ND
KH015	ND	444	KPC-3	2 (S)	1 (S)	ND	0.25 (S)	ND	ND	ND
KP020	ND	526	KPC-2	2 (S)	1 (S)	8 (S)	1 (S)	ND	1 (S)	ND
KC006	ND	560	KPC-2	2 (S)	16 (R)	ND	4 (R)	ND	ND	ND
KH027	ND	971	KPC-3	2 (S)	1 (S)	ND	2 (R)	ND	1 (S)	ND
KR021	ND	1198	KPC-2	2 (S)	1 (S)	ND	0.25 (S)	ND	1 (S)	ND
KC002	ND	1377	KPC-2	2 (S)	16 (R)	32 (R)	4 (R)	256 (R)	ND	20 (R)
KP057	ND	1533	KPC-2	64 (R)	16 (R)	ND	0.25 (S)	ND	0.5 (S)	ND
KH006	ND	1661	KPC-3	2 (S)	16 (R)	ND	4 (R)	ND	ND	ND
KL048	ND	1703	KPC-2	2 (S)	1 (S)	ND	0.25 (S)	ND	1 (S)	ND
KP019	ND	1704	KPC-2	16 (S)	16 (R)	32 (R)	1 (S)	64 (R)	ND	320 (R)
KC003	ND	1705	KPC-2	2 (S)	1 (S)	32 (R)	4 (R)	ND	2 (R)	ND
KP025	ND	1706	KPC-2	16 (S)	1 (S)	32 (R)	4 (R)	ND	4 (R)	ND
KC004	ND	1707	KPC-2	2 (S)	16 (R)	16 (S)	1 (S)	ND	1 (S)	ND
KP007	ND	1708	KPC-2	8 (S)	1 (S)	32 (R)	4 (R)	ND	ND	ND
KP056	ND	1887	KPC-2	64 (R)	16 (R)	ND	0.25 (S)	ND	0.5 (S)	ND
			**n**	77	76	28	77	10	58	10
			**Susceptible**	57	32	5	21	0	19	0
			**Resistant**	20	44	23	56	10	39	10

ND, No data. All bacterial isolates are part of the strain collection of the Grupo de Investigación en Microbiología Básica y Aplicada (MICROBA) and were collected in the city of Medellin, Colombia. Identification and susceptibility tests were performed using the semi-automated method VITEK® 2, genes that encoded carbapenemases were identified by PCR and sequenced to determine gene-variants. Molecular typing was performed using Multilocus sequence typing (MLST). Isolates whose susceptibility interpretation was intermediate were considered resistant. The interpretation of the minimum inhibitory concentrations (MIC) was done according to CLSI M100 Performance Standards for Antimicrobial Susceptibility Testing S25 and EUCAST clinical breakpoints v 4.0.
